# Delayed Diagnosis of a Pyloric Web Causing Gastric Outlet Obstruction in a 13-Month-Old Girl

**DOI:** 10.1055/s-0041-1723017

**Published:** 2021-03-03

**Authors:** Mohammed Elifranji, Jisha Sankar, Israa Abdelrasool, Guy Brisseau

**Affiliations:** 1 Department of Surgery, Sidra Medicine, Doha, Qatar; 2 Department of Pediatric Surgery, Hamad Medical Corporation, Doha, Qatar; 3 Department of Gastroenterology, Sidra Medicine, Doha, Qatar; 4 Department of Pediatric Surgery, Sidra Medicine, Doha, Qatar

**Keywords:** gastric outlet obstruction, pyloric web, atresia

## Abstract

Pyloric web is a rare cause of gastric outlet obstruction. Classical pyloric web can be diagnosed by obtaining a patient history, physical examination, and plain abdominal X-ray, whereas a perforated web leads to incomplete intestinal obstruction. Delayed diagnosis is rare, and the definite diagnosis is made by upper endoscopy. In this report, we report a case of a girl in whom a pyloric web was diagnosed at the age of 13 months.

## Introduction

Gastric outlet obstruction can be caused by prepyloric or pyloric abnormalities. Most of the cases reported in the literature refer to the gastric antral web as a cause of gastric outlet obstruction. 1 Pyloric web, the most common type of pyloric atresia (PA), is diagnosed during the neonatal period with nonbilious vomiting. Its delayed detection is very rare. 2 Hence, only a few cases report on pyloric web as a cause of gastric outlet obstruction in infancy. 1 We report a case of an infant with a delayed diagnosis of pyloric web, as well as the challenges in the diagnosis and management.

## Case Report

**Fig. 1:**
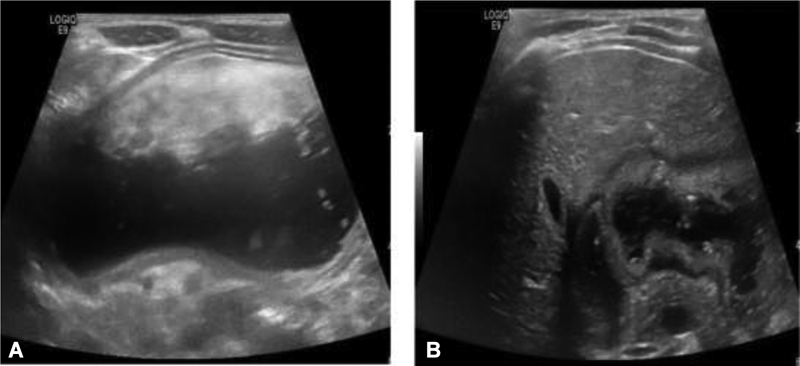
Ultrasound of the abdomen showing a grossly distended stomach. ( **A** ) and normal pylorus without thickening ( **B** ).

The patient was a 13-month-old girl who was born full term with a birth weight of 3.1 kg. Her symptoms started with nonbilious vomiting on the second day of life; however, her growth and weight were not affected. After 6 months of age, her symptoms progressed, and occasional coffee ground vomiting started in addition to a plateau in her weight. Therefore, different milk formulas were tried without much improvement of the vomiting. Her weight was on the third percentile, and the blood tests were negative for food allergens, *Helicobacter pylori* , and celiac disease, but positive for occult stool blood. The abdominal ultrasound showed a distended stomach and a normal-looking pylorus ( Fig. 1A, B ).

**Fig. 2:**
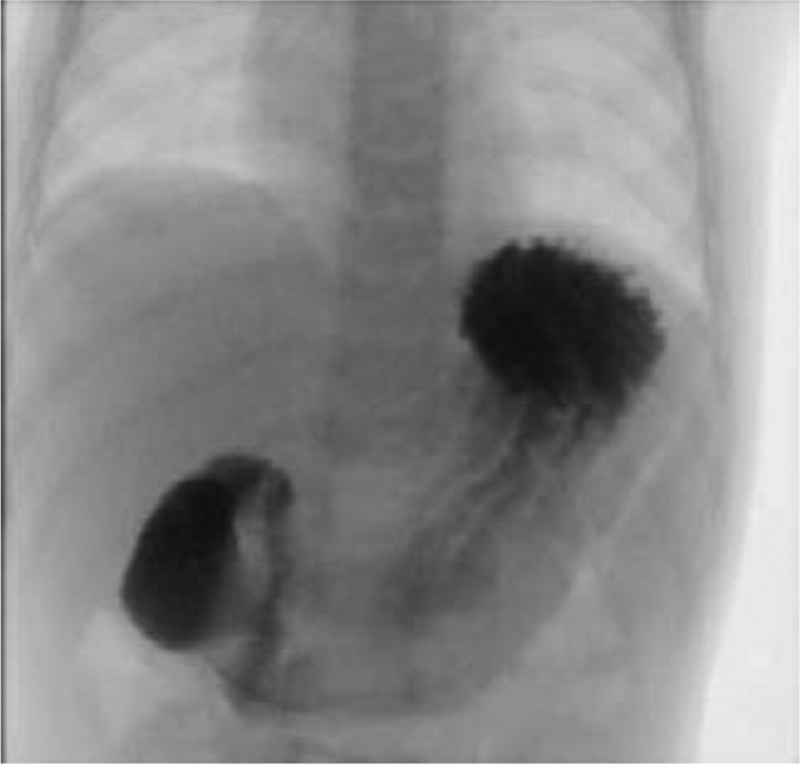
Upper gastrointestinal contrast showing delayed passage of contrast from the stomach into the duodenum.

Consequently, upper gastrointestinal (UGI) contrast study showing delayed gastric emptying without anatomical filling defect ( Fig. 2 ).

**Fig. 3:**
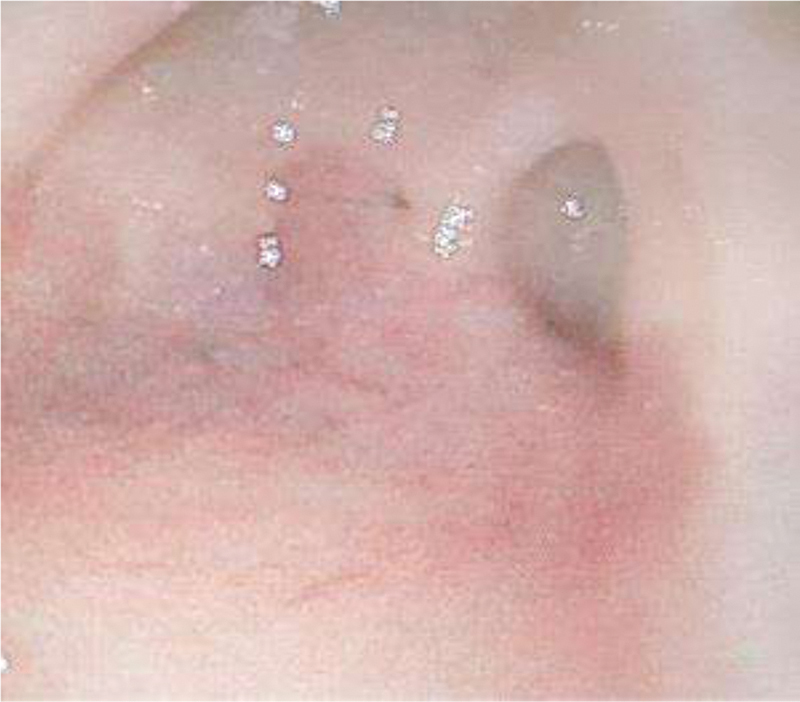
Esophagogastroduodenoscopy showing a pyloric web with a tiny hole and inflammation in the stomach.

Therefore, the child was treated conservatively for delayed gastric emptying and symptomatic gastroesophageal reflux. However, she continued to vomit beyond a certain volume of food especially solid food. Thus, an esophagogastroduodenoscopy was performed, which showed a pyloric web with a pinhole opening in addition to a diffuse inflammation in the stomach and lower esophagus. Several attempts were made to cannulate the pylorus but with no success ( Fig. 3) .

After a week adjustment of the malnourished state through parental nutrition through a peripherally inserted central catheter line, the patient was taken for operative exploration through a right upper quadrant transverse incision. The pylorus was delivered and looked thickened, and a full-thickness longitudinal incision on the anterior surface of the pylorus was made and a pyloric web was detected. A partial excision of the web was performed leaving the posterior wall of the pylorus intact followed by a Heineke-Mikulicz pyloroplasty. The patient was started on oral feeds after 48 hours with low volumes, which were gradually increased to achieve full oral feeding on postoperative day 5.

## Discussion

Pyloric web is the most common type of PA, which in itself is a very rare pathology. 2 PA constitutes approximately 1% of all intestinal atresias, with an incidence of approximately 1 in 100,000 live births. 3 4 Although the developing mechanism is not clear, some hypotheses suggested a susceptibility, genetic transition, recanalization, and vascular damage in the embryonic period. 5 6 From an anatomy point of view, PA has been classified into three types: a pyloric membrane/web (type 1: 57%), a solid cord (Type 2: 34%), or a gap between the stomach and duodenum (type 3: 9%). 3 7 It can also be classified into isolated PA (35%), PA associated with epidermolysis bullosa (40%), and PA associated with other anomalies (25%). 8

Therefore, the presenting symptoms and time of diagnosis can vary between cases according to the degree of obstruction and associated comorbidities. Prenatal diagnosis may be possible in cases with a dilated stomach, absence of a double bubble sign, and presence of polyhydramnios, as reported in 50% of cases in the last trimester. However, the findings were not consistent due to the physiological variation in the fetal stomach size and infrequent appearance of polyhydramnios. 9 10 11 On the other hand, the presentation after birth can vary from nonbilious vomiting, failure to thrive, and abdominal distension. Delayed presentation is possible and could be due to a partial obstruction causing a subtle course of the disease, as well as later presentation of the symptoms, absence of primary health care facilities, and possible ignorance of this rare entity, as reported in Kansra et al's study that showed the median age at the onset of symptoms was 6 months (1 day to 36 months) and the median age at presentation to the hospital was 7 months (1 day to 44 months) with a 5-month mean delay between them. 12

The diagnosis of PA cases is straightforward and can be achieved through patient history, physical examination, and plain abdominal X-ray. An exception is a perforated web causing a partial intestinal obstruction. The diagnosis can be delayed, but radiological, ultrasonographic, and endoscopic investigations can be helpful. 6 Even though it is challenging to make an accurate diagnosis, which can be delayed even with the use endoscopy or UGI series, such an entity is rare and cannot be easily managed by a clinician who has no previous knowledge about it. Such patients including our case have been treated for pyloric spasm with hyperacidity. 13 14 Moreover, the partial improvement with the H _2_ blocker suggests that feeding intolerance with occasional coffee ground vomitus could be due to peptic ulcer disease in spite of being not common compared with older children. Hence, a high index of suspicion is required in patients presenting with nonbilious vomiting and failure to thrive as they may be wrongly diagnosed and treated for gastroesophageal reflux or peptic ulcer disease.

The treatment for PA is surgical and depends on the type of atresia. 7 15 16 The recommended procedure for type 1 is an excision of the web and a Heineke–Mikulicz pyloroplasty and for type 2 is the excision of the pylorus with end-to-end gastroduodenostomy. However, Dessanti et al 16 described a pyloric sphincter reconstruction: “gastroduodenal mucosal advancement anastomosis.” For type 3, the treatment is gastroduodenostomy.

The endoscopic treatment is not clear for PA, but it may be considered for antral web when endoscopic intervention is feasible. If the mucosal structure of the antral web is uniform without major vessels or muscular or serosal layers and the membrane is tense and consistent with perpendicular insertion, then endoscopic transection is possible. 17

In this study, our case had an isolated, incomplete type 1 atresia and therefore presented with subtle and nonspecific symptoms that waxed and waned over a period of time and made the diagnosis challenging. Although endoscopy was useful for making the diagnosis, it was not effective for treatment due to the failure to cannulate the pyloric web hole. Thus, surgical intervention was commenced, and a modified technique was performed to repair the pyloric web. The incision of the web was accomplished followed by a Heineke–Mikulicz pyloroplasty. Our patient had an uneventful postoperative course and went home on day 5 after tolerating oral feeding. Furthermore, it is deemed that the excision of the web can cause avoidable complications, such as injury to major blood supply or nearby biliary drainage system, and achieve a similar outcome when simply incising it. However, further prospective studies are needed.

In conclusion, pyloric web is a rare entity of gastric outlet obstruction and can present with nonspecific symptoms that can cause a delay in diagnosis. Thus, a high index of suspicion in addition to an UGI endoscopy can help achieve a definite diagnosis. Moreover, surgical intervention remains the best treatment.

## Notes

New Insights and the Importance for the Pediatric Surgeon Pyloric web is a rare entity of gastric outlet obstruction and can present with nonspecific symptoms that can cause a delay in diagnosis. Thus, a high index of suspicion in addition to an upper gastrointestinal endoscopy can help achieve a definite diagnosis. Surgical intervention remains the best modality of treatment.
